# Nutrition security in Malawi: navigating climate shocks, food insecurity, and malnutrition in the 2025 landscape—narrative review

**DOI:** 10.3389/fnut.2026.2004735

**Published:** 2026-10-26

**Authors:** Julius Niyibizi, George Chikondi Samu, Alieu Sam, Noreen Clara Ng’ambi, Emmanuel Nizeyimana, Maria Yulita da Silva Ximenes, John Mugisha

**Affiliations:** 1 Southern Medical University, Guangzhou, China; 2 Rwanda Implementation Science and Health Systems Lab, Kigali, Rwanda; 3 Ministry of Health and Sanitation, Freetown, Sierra Leone; 4 Ministry of Health, Lilongwe, Malawi

**Keywords:** climate shocks, food insecurity, malnutrition, nutrition security, stunting

## Abstract

**Background:**

Malawi’s nutrition security is undermined by reliance on smallholder rain-fed agriculture, recurrent climate shocks, poverty, gender inequities, weak infrastructure, and fragmented governance. Recent estimates indicate that approximately 37% of children under 5 years of age experience stunting, while projections for the 2025/2026 lean season suggest that up to 4 million people may face acute food insecurity.

**Methods:**

This narrative review synthesized evidence from peer-reviewed literature and relevant institutional reports to examine the relationship between climate shocks, food insecurity, and nutrition outcomes in Malawi. Literature published from 2000 onwards was considered, with particular attention to evidence from January 2023 to November 2025 relevant to the country’s 2025 nutrition security landscape.

**Findings:**

Climate shocks were associated with approximately 20% maize production losses, increased food prices, and reduced dietary diversity. Available projections suggest that under adverse climatic and socioeconomic scenarios, stunting and wasting could increase to approximately 38–40 and 12%, respectively, by 2027, particularly among vulnerable groups. Climate-smart agriculture, biofortification, and cash transfers show potential but remain constrained by limited funding and coordination challenges.

**Conclusion:**

Strengthening nutrition security in Malawi requires integrated approaches linking climate adaptation, resilient food systems, nutrition interventions, and multisectoral coordination. Long-term progress will depend on sustained investment, improved implementation capacity, and policies that address the structural drivers of vulnerability.

## Introduction

At a multidimensional level, nutrition security involves more than the mere availability of food: it also includes access to and utilization of food, and their long-term stability (1, 2). This comprehensive framework is critical not only for protecting the health of Malawian populations, but also to prevent economic shocks in high-risk groups such as children, pregnant women and people living with HIV (3). Malnutrition has resulting long-term effects in this context by hampering physical growth, leading to heightened risk of diseases and reducing cognitive capacity as well as general wellness (4). Consequently, securing nutrition is a cornerstone to protecting human capital and supporting sustainable development in Malawi (1, 5).

Malawi is an agrarian economy highly reliant on rain-fed agriculture, with substantial dependence on staple crops such as maize. This reliance renders the country particularly exposed to climate perturbations such as droughts, unpredictable rainfall and cyclone events, which disrupt production systems (6, 7). The high rate of smallholder farming also exacerbates this challenge, resulting in many households being vulnerable to agricultural shocks (7). Historical and recent evidence underscored in the Malawi Demographic Health Survey, World Food Programme reports continue to reflect long-standing challenges of food insecurity, stunting and wasting that warrant immediate attention for holistic nutrition security strategies (3, 7).

There is a clear correlation between climate shocks, food insecurity and malnutrition. Food systems are disrupted by extreme climate events through effects on agricultural productivity, changes in food market dynamics leading to increased food prices and decreased access to sufficient and nutritious foods (1, 5). Such disturbances have short-term implications, including increased prevalence of child wasting, but also have long-term ramifications such as stunting and impaired cognitive development (3, 4). The synergized impact of poverty and environmental uncertainty has rendered the promotion of nutrition security an urgent public health agenda in Malawi (1, 5).

Considering the current climate and socioeconomic context, Malawi entered the 2025 period with persistent vulnerabilities in food and nutrition security. Projections indicate that about 4 million people could suffer from food insecurity in 2025 delineating the major need for dynamic measures and efficient policies (6). This review seeks to uncover the interactions between climate shocks, food insecurity and nutrition outcomes in Malawi. In seeking to do so, this will involve a critical examination of current interventions, climate adaptation activity and social safety nets that have already been put in place in an effort to address such challenges (5). The evidence synthesized by this review is anticipative of informing the development and implementation of resistant, climate-adaptive and food secure strategies that secure both food and nutrition in Malawi, which would lead to sustainable development despite climatic adversities (1, 4).

### Methodology

This narrative review brings together what is known from the literature about the complex links between climate shocks, food insecurity, and poor nutritional outcomes (including undernutrition) to inform nutrition security strategies in Malawi with a forward-looking focus on the 2025 setting. Narrative rather than systematic reviews take a more flexible, interpretive stance to provide an overview of the evidence base that can influence policy and practice, drawing on a range of evidence whilst allowing for any gaps (8, 9). The method utilized well-established narrative synthesis—thematic analysis and critical reflection—rather than rigorous meta-analysis.

The review addresses the broad research question: ‘How do climate shocks heighten food insecurity and malnutrition in Malawi and what adaptation strategies can be implemented to improve nutrition security by 2025?’. The purposive literature search was conducted up to 30 November 2025 to identify evidence on the relationships between climate shocks, food insecurity, and malnutrition in Malawi. Literature published from January 2000 onwards was considered to capture long-term trends in climate vulnerability, agricultural systems, food insecurity, and nutrition outcomes. Particular attention was given to evidence published between January 2023 and November 2025 to incorporate recent developments, including post-Cyclone Freddy impacts, El Niño-related drought conditions, and emerging projections relevant to Malawi’s 2025 nutrition security landscape. Several electronic databases were accessed, including PubMed / MEDLINE, Scopus, Web of Science, Google Scholar as well as grey literature databases such as the World Food Programme (WFP) reports, Malawi Demographic and Health Surveys (MDHS) and the Food and Agriculture Organization (FAO) archives. Search terms were combined using Boolean operators and included combinations of the following concepts: “Malawi” AND (“climate change” OR drought OR flood OR cyclone OR “climate shock*”) AND (“food insecurity” OR “nutrition security” OR malnutrition OR stunting OR wasting). Additional searches included intervention-related terms such as “climate-smart agriculture,” biofortification, social protection, and early warning systems. Results were filtered using Boolean operators (e.g., AND, OR) as well as truncation (e.g., climat* shock*) resulting in approximately 1,200 first records.

### Eligibility criteria

Eligible sources included peer-reviewed articles, government reports, and organizational evaluations published in English from 2000 onwards. Included studies were required to address Malawi’s agrarian context, vulnerable populations (including children under 5 years and pregnant women), or the relationship between climate shocks, food systems, and nutrition outcomes.

### Exclusion criteria

Exclusion criteria included non-Malawi-specific articles, non-empirical opinion pieces, and duplicates. Titles and abstracts were screened by the lead author according to predefined eligibility criteria. A second reviewer independently screened a randomly selected 10% sample of records as a quality assurance procedure to evaluate consistency in applying inclusion criteria. Disagreements were resolved through discussion until consensus was reached. Following full-text assessment, 58 sources were included for narrative synthesis. Through this iterative process, a balance of public health, agriculture, economics and environmental science disciplines were represented.

### Data extraction

Data extraction highlighted specific themes related to the structure of the review, including: agriculture vulnerabilities; climate risks; food access dynamics; nutritional consequence; intersecting vulnerabilities; and policy response. Quality assessment of included studies was conducted using narrative indicators: methodological quality (e.g., study design, sample size), relevance to 2025 forecasting, and consideration of equity (e.g., gender, rural–urban dissimilarities). Synthesis comprised of thematic coding and narrative synthesis, rooted in conceptual frameworks such as the UNICEF nutrition framework and climate-food security models (1, 10), prioritizing analytic depth over quantitative synthesis. No dedicated meta-analysis was conducted, analytic depth being pursued rather than quantitative synthesis.

### Malawi’s agriculture, economy and nutrition status

The Malawian economy is largely an agro-based economy, with agriculture accounting for approximately 30% to the country’s gross domestic product (GDP) and employing much of its workforce, particularly in the rural areas (11). Agricultural land use is dominated by rain-fed systems and staple crops predominantly maize, which plays an important role in food security of the country (11). Over 70% of the agricultural production is carried out by smallholder farmers who frequently lack contact with modern agricultural technologies, quality inputs and sound market links (12). This reliance on traditional farming also renders the sector susceptible to climatic shocks including droughts and untimely rains, with wealth effects observed to vary across rural households (11).

Problems in Malawi’s agricultural sector are compounded by infrastructure weaknesses such as poor irrigation systems, inadequate rural roads, and insufficient storage facilities that contribute to production losses (11). Furthermore, soil depletion and non-mechanization (which reduces yield improvement) increase vulnerability with the high dependence on maize: a crop that is also sensitive to climate shocks and changes in market price (12). These weaknesses do not only erode the resilience of food systems but also perpetuate socio-economic inequalities given that rural communities often have few income-generating options and greater likelihood of being food insecure (11).

Malnutrition remains a major public health challenge in Malawi, with recent estimates indicating that approximately 37% of children aged less than 5 years are stunted and about 10% are wasted (12). These effects are inseparable from the nation’s underdeveloped agricultural productivity and economic inequality. These factors, compounded by insufficient access to a variety of foods, increasing food costs from seasonal shortfalls and lack of optimal feeding practices impact negatively on the nutritional quality of diets for those most vulnerability (9). The fact that rural household diets rely heavily on maize implies the climate-stress-induced inability to access other food sources increases risks of micronutrient deficiencies and poor growth outcomes (12, 13). Thus, the relationship we see between Malawi’s agrarian economy, infrastructural limitations and constrained adaptive capacity cycles economic poverty with poor nutrition outcomes.

The agricultural and economic setting of Malawi is largely based on a smallholder, rain-fed farming system with a dual focus on maize as its chief crop; this has created an environment where the food insecurity and poor nutritional status are intertwined. The prevailing socio-economic concerns of rural sustenance communities and the pressing infrastructural limitations along with the vagaries in climate patterns make it imperative to effect composite interventions that simultaneously address issues relating to sustainable agricultural practices as well as dietary diversity and nutrient intensity (12).

### Climate shocks and environmental risks

Malawi is growingly affected by various climate shocks which are greatly deteriorating its agricultural productivity and general food security (8). Droughts remain one of the most common hazards, causing acute water shortages that result in immediate maize production losses the country’s main staple crop. Maize is very sensitive to water stress and has its yields reduced substantially during dry periods, which affects the availability of food for many Malawians (2). Erratic in the pattern of rainfall further complicates this situation as it interferes with planting calendars and erodes predictability around an agricultural calendar that underpins the life of smallholder farmers (2, 14).

Floods are also a significant threat in Malawi, especially in central and southern regions where intense rainfall saturates extensive farmland, and destroys essential infrastructure including roads and storage facilities resulting in displacement of rural communities (14). The loss of crops from flooding is compounded by the length of time infrastructure takes to repair, which can drive longer periods of food insecurity. Recent weather shocks, including events like Cyclone Freddy in 2023, triggered additional crop losses and increased food insecurity by exacerbating flooding and interrupting local markets (14). The combined effects of these climate extremes have not only reduced current agricultural productivity, but also weakened the robustness of food systems which are already fragile with high dependencies on rain-fed agriculture.

These climate shocks are eroding Malawi’s food security and increase its long-term environmental risks (15). Continual over-cultivation, excessive deforestation and improper soil management practices translate into soil degradation that diminish the capacity of land to support healthy agricultural returns (14, 16, 17). Environmental degradation that arises as a result of the aforementioned factors also includes loss of biodiversity and decreased ecosystem services necessary for agriculture in the long run (18). Further projections suggest that increased temperatures and greater climate variability will amplify these vulnerabilities by heightening droughts and extreme precipitation events, which would further undermine the country’s already fragile food systems (19). Sexual minorities, especially those living in rural parts of the southern and central regions, are disproportionately affected by exposure to these environmental hazards. Small holder farmers with little access to irrigation and adaptive agriculture mechanisms not only suffer economic hardship but are most at risk for malnutrition. Food availability uncertainty directly affects the poorest households, for which low dietary quality and diversity are leading to child-, women-and elderly undernutrition (18, 20). Therefore, the interrelationship between climate shocks and environmental degradation stimulate a vicious cycle of food insecurity and malnutrition and highlight the critical role for adaptive strategies that enhance resilience against such adverse conditions in Malawi agricultural systems.

### Food insecurity: availability, access, and price/market dynamics

Food insecurity in Malawi is a multifaceted problem comprised of those challenges related not only to the availability, but also access and market uncertainty that combined to erode the nutritional status of its peoples. Climate-induced disruptions severely impact food availability. Droughts and unseasonal rainfall patterns, have adversely affected maize the staple crop, and other critical harvests time-and-again (21). This shock is most severe during the lean pre-harvest months when agricultural production declines significantly and widespread food insecurity results. This then creates shortages putting government and humanitarian organisations reliant on importing food aid to plug the gap between local production and consumption, leading to a cycle of repetition (21). Forecasters predict that the seasonal disruptions will likely get worse as climate change increases weather unpredictability and places more pressure on food supplies in an already fragile setting.

Food access is inherently uneven in Malawi, with enormous variations between urban and rural spaces. Rural households that are predominantly engaged in subsistence farming and have lower household income are more disadvantaged especially in the absence of infrastructure such as good road network and few market facilities (22). This infrastructure gap increases the cost and degrades the predictability of food distribution, in particular during the hungry season when local supplies run low and market access is even more constrained (22). Moreover, socio-cultural factors such as inequitable distribution of food acquisition and preparation between women and men, high family sizes also compromise the ability of vulnerable groups to ensure a nutritionally adequate diet.

Food prices and market forces also conspire to make Malawi even more food insecure. Drought induced crop failure is a commonly reported phenomenon which lead to the sudden shrinking of food supply followed by increase in prices (11). These changes are compounded by high transport costs and market uncertainty, adding further strain on poor households, who devote a notably large average share of income to food (8). In the absence of strong government interventions (e.g., efficient subsidies or price stabilization mechanisms), price inflation reduces household purchasing power and access to a diversified and nutritious food basket. The result is that market volatility not only undermines local food systems but also exacerbates the vulnerability of the rural population and by extension of all other marginalized groups.

### Malnutrition and nutritional outcomes

Malnutrition in Malawi constitutes a complex public health concern, the related underpinning factors largely include food insecurity, poverty and poor feeding habits. Chronic undernutrition is largely evident through stunting, wasting and underweight, and recent evidence suggests 37% of children under 5 years are reported to be stunted while around 10% experience wasting (22). In addition, deficiencies in micronutrients are widely prevalent and they also have a major detrimental impact on health and development of both children and adults (23). Positive but indirect effect is played by maternal malnutrition on the adverse birth outcomes, such as low birth weight, which then leads to a continued inter-generational cycle of malnutrition and economic vulnerability (24).

Children under 5-years of age are disproportionately affected by malnutrition in Malawi, with resultant impacts on cognitive development, immune function and learning (23). At-risk populations, such as lactating and pregnant women and the elderly also suffer; nutritional deficiencies in women do not only put their health at risk but negatively affects fetal development as well early childhood growth, whilst the elderly generally experience a low variety of diets with poor access to food (24, 25). These nutritional deficits are intensified by seasonality, with the lean period coinciding with a decrease in food availability at household level in many rural areas. The result is an acute malnutrition burden, wasted and underweight, exposing a vital association between seasonal food insecurity and nutrition outcomes (22).

Apart from the immediate health consequences, malnutrition has far-reaching socio-economic implications for Malawi’s developmental prospects in the longer term. Early childhood nutritional deficits are associated with poor cognitive and physical development in early life, and lower educational achievement and productivity as adults (26). This reduction in human capital perpetuates poverty and food insecurity cycles by malnourished people being unable to contribute productively to the economy to maintain the status quo under which wide ranging nutrition deficiencies exist (27). And the economic costs of malnutrition are high, including increased health-care expenditures and decreased workforce productivity (23). Table 1 presents key nutrition security indicators in Malawi and their projected trends toward 2025. The data indicate a likely worsening of stunting, wasting and overall food insecurity, largely driven by ongoing climate shocks and economic pressures.

**Table 1:** Observed nutrition security indicators and projected scenarios relevant to Malawi’s 2025 landscape.

Indicator	Observed estimate	Future estimate	Key drivers	Source(s)
Stunting (children <5 yrs)	37%	38–40%	Climate shocks, poverty	MDHS, Kodish et al. (36)
Wasting (children <5 yrs)	10%	12%	Seasonal food shortages	WFP, Chilanga (24)
Food insecure population	~3 million	4 million	El Niño, high prices	Araya et al. (6), IPC
Maize production reduction	10–15% (post-Freddy)	20%	Droughts, floods	Rana et al. (11), FAO
Micronutrient deficiency	Iron: 50%; Vit A: 70%	Rising 5–10%	Limited dietary diversity	Global nutrition report

Quantitative indicators presented in table should be interpreted according to their evidence type. Observed values describe reported population-level estimates, whereas projected values represent scenario-based estimates influenced by assumptions regarding climate conditions, economic trends, and policy responses.

### Intersecting vulnerabilities and special populations

Overlapping vulnerabilities in Malawi form a dynamic mosaic of risks that unequally burden certain population groups, exacerbating the negative effects of food insecurity, malnutrition, and climate shocks on already vulnerable households. Complications faced by children under five, pregnant and lactating women, the elderly, people living with HIV/AIDS (PLWHA) as well as smallholder farmers which compromise their nutritional and general well-being is at the center of many problems facing the country.

Infants and young children are particularly at risk as they are very susceptible to malnutrition and its lasting effects. Stunting and wasting are associated with short stature, impede growth and development, interfere with cognitive development and challenge future educational attainment and economic productivity (28). Maternal diet contributes to the health of the child; poor maternal and good nutrition is correlated with low birth weight and infant mortality and perpetuates intergenerational malnutrition cycle (29). Chronic exposure to food insecurity, especially during lean seasons marked by reduced crop productivity driven by climate-induced shocks, compounds these deleterious effects. This emphasizes the role of specific nutrition interventions that tackle maternal and child health together (28, 29).

Malawian pregnant and lactating women have a severe malnutrition burden that impairs their health and that of their children. Food insecurity and poor maternal nutrition increase the risks of LWB, preterm delivery and maternal anemia as well incurring greater risk for infant mortality (28). The additional demands for nutrition in pregnancy and lactation, especially in essential micronutrients like iron and folic acid, often remain unfulfilled among women living in food insecure conditions (30). As a result of poor nutrition during these critical periods, maternal health is compromised and with it the potential for optimal infant growth and development such that cycles of vulnerability pass from one generation to the next (28).

Older people in Malawi represent a second group whose nutritional status is doubly jeopardized due to interacting vulnerabilities. Food insecurity is a chronic experience among many of the elderly because of limited mobility, declining income as well as aging-related physical struggles (31). Lack of appropriate healthy food adds to ongoing chronic health problems, increases susceptibility to infections, and degrades quality of life. Economic vulnerability, reduced physical capacity, and lack of focused social safety nets may render older adults on the margins of nutrition and health responses despite considerable risks (31).

Individuals with HIV/AIDS (PLWHA) are especially hard-hit by food insecurity, which further compounds the impact of the disease through poor nutrition and unpredictable access to food. In addition to wasting and malnutrition, dietary deficiency is related to the effectiveness of ART regimens because of complications associated with drug-food interactions as well as issues with treatment compliance (32). The immunosuppressed state of PLWHA coupled with nutritional deficiencies can result in a vicious cycle, in that poor nutrition further compromises health and susceptibility to OAIs. There was need to have holistic approach that integrate food security intervention with the healthcare of these population in order to not only improve nutrition but also health status (32).

Smallholder farmers—the backbone of Malawi’s agrarian economy—are also living in a world of vulnerability and resilience. Their reliance on rain-fed agriculture and limited diversity of staple crops, especially maize, makes them particularly vulnerable to climate shocks including droughts and floods (33, 34). These negative climatic occurrences result in harvest losses which reduce both household food stocks and incomes, thereby impacting nutrition. This gets compounded by a lack of access to farm inputs, modern irrigation technologies and effective marketplaces, which yokes smallholder farmers into an economic cycle that further reduces productivity while significantly increasing food insecurity and malnutrition at household levels (33). Moreover, the limited resources of those farmers further limit their capability to invest in adaptation practices that could help reducing the impact of future climate shocks—and this overall contributes to increasing the vulnerability of the rural food system.

### Resilience, adaptation, and policy/program responses

Resilience, Adaptation and Coordinated Policy are Key Components to Malawi’s response to Food insecurity, malnutrition and climate shocks. The agricultural sector adaptive capacity has increasingly become a priority for government agencies, non-governmental organization (NGOs), and international organizations as they struggle to enhance resiliency to climate variability at the same time as improving nutrition outcomes among vulnerable populations (33). These projects do not just consider immediate relief but also build up sustainable structures for the future. This discussion transcends current strategies and programs, considers their efficacy, and identifies areas for improving climate adaptation’s coherence with nutrition-sensitive agriculture.

Climate-smart agriculture is a key component of Malawi’s adaptation strategy. Farmers have been urged to adopt drought-resistant crop varieties, improved irrigation technologies and better agronomic practices in attempts to reduce vulnerability to erratic rainfalls and perennial droughts (35). Diverse agriculture, combining livestock, fisheries and nutrient-rich and biofortified (vitamin A maize and iron-rich beans) crops to reduce the risk of over-reliance on one staple such as maize is also in order (36). Moreover, early warning systems and climate information services as well as weather-indexed insurance products have been developed to support farmers in the preparation for, and response to climate shocks. Such tools contribute to stabilizing production periods and mitigate uncertainty following environmental hazards so as to enhance food security in households (35, 37).

In parallel with these efforts, nutrition-sensitive agriculture has been advocated to address food insecurity and malnutrition at the same time. This holistic model highlights the necessity to mainstream nutrition priorities into agriculture policies in order to promote the growing of micronutrient-rich crops—i.e., beans and vegetables as well as dietary diversity (36, 38). Biofortification programs are key as they seek to improve the micronutrient composition of staple crops consumed by rural populations, and they correspondently address directly the vitamin and mineral deficiencies that are prevalent in Malawi (38, 39). By connecting more efficient practices in farming with nutritional results, such endeavors help to lower malnutrition and at the same time build up economic stability within rural society.

These resilience-building efforts have also been supported by social protection programs in Malawi. Cash transfers, food assistance and school feeding programmes have been established to mitigate the effects of climate shocks and economic instability on those most at risk. For example, the Malawi Social Cash Transfer Program, designed to reach households that include pregnant women, children, and older people, has been found to have beneficial effects on household food security and nutritional status (40, 41). Yet such initiatives face obstacles in geographical reach, resource availability and intermittent as opposed to coordinated policy backing. Scaling-up successful pilots while ensuring the long-term sustainability of these social protection measures should continue to be a priority.

Concurrently, response by the health system is also key to addressing malnutrition head-on. Child and maternal supplementation, targeted therapeutic feeding programmes and community-based nutrition services are now part of primary health care. These interventions have enhanced the ability of health workers to detect, treat and prevent malnutrition, and as a result stunting and wasting rates are lower (36, 42). However, there are still difficulties in systematically mainstreaming nutrition into health and agricultural policies. Poor level of coordination across sectors has constrained effective delivery of multisectoral interventions, calling for coherence between climate, agriculture and nutrition policy frameworks (42, 43).

However, while these diverse approaches have made substantial advancements in policy and programmatic responses, there are still important areas of neglect. Lack of funds, sectorial dispersion and coordination problems have in many cases prevented among others from reaching scale (43). In addition, although there have been a number of pilot projects showing positive results there remains the need for sustained policy investment to ensure these gains are embedded in long-term organizational funding cycles (44). Multi-sectoral approaches that cut across food security, nutrition and climate change should be scaled up, along with adaptive measures which mainstream agricultural innovation into health and social protection programmes. Future policy should support development of civil servant capacity to advance resilient transitions and utilize evidence-based planning to bridge the divide between global nutrition movements and local adaptive strategies (36, 38).

### Challenges and emerging issues

The problems facing, and the prospects confronting, nutrition security in Malawi are multi-faceted as environmentally, economically, socially, and institutionally related physical factors overlap to exacerbate food insecurity and malnutrition. Their complexities are discussed in detail later, illustrating the importance of integrated multisectoral interventions.

Malawi is faced with the challenges of climate change and environmental vulnerabilities. The nation is experiencing a rise in both the number and magnitude of droughts, changes in precipitation patterns, and floods which have an adverse effect on agricultural output (21). These shocks result in erratic crop yields, and the ensuing food price increase aggravates food insecurity among smallholder farmers and rural communities (21, 45). Chronic environmental degradation represented by soil erosion, deforestation, and water shortage also adds to the difficulty of maintaining agricultural productivity in the longer term (46). These environmental constraints call for the use of adapted farming practices such as climate-smart agriculture and improved water management techniques (47).

Economic constraints and volatile markets compound the issue of nutrition security in Malawi. The country is affected by inflation, drop in currency value and large price shortages which interfere with ease of access and affordability for foods (48). Global economic trends, involving the price of food and fuel, as well have an effect on Malawi’s ability to import some basic food stuffs that in turn worsens local food shortages when domestic production falls short (10). Poorly developed agricultural market systems deny smallholder farmers access to lucrative markets coupled with lack of infrastructure and expensive transport costs, which in turn are compounded by information availability (49, 50).

Gender and social inequities play an important role in shaping nutritional status. The prevalence of gender inequality in Malawi translates to a limited women’s access to land, agricultural inputs, and financial resources (which leads to) poor intra-household food distribution and child nutrition (51). Cultural values regarding the use of maize and certain child feeding practices for instance, these prevent diversity in diets and help to keep malnutrition sticked (51, 52). Added to these social constraints is the burden of household poverty which limits access to healthy foods and quality healthcare (53).

There are also chinks in the system for public health and nutrition that further hinder attaining NS. Deficiencies in medical infrastructure, particularly in rural locations; shortages of trained health staff; and poor nutritional elements in the provision of healthcare services limit a more appropriate reaction to malnutrition (54). The nexus of malnutrition and infectious disease prevalence underscore the imperative to implement combined nutritional, health and nutrition education interventions (54).

New considerations of technology and innovation are a double-edged sword for nutrition security. These can also include digital agro-technology such as the use of precision agriculture tools or early warning schemes which improve farmers’ resilience to climate variability and foster increases in productivity (47). Movement toward value added agricultural products via local food processing ventures suggest a new form of Malawian food systems although scaling up and just access remain critical issues (55). New technologies such as ICT enabled market information services help reduce market inefficiencies and enable smallholder farmers cope with economic threats (50). Recent advances in artificial intelligence, including large language models such as ChatGPT, are increasingly being explored in global health systems to support health information delivery and decision-making processes. Evidence from a recent global cross-sectional study suggest their potential application in healthcare informatics, which could be adapted to strengthen nutrition surveillance and early warning systems in resource-limited settings such as Malawi (56).

The weaknesses in policy and institutional frameworks are also an attack on our efforts to enhance nutrition security. Fragmented coordination between government institutions, low political commitment and inadequate investment in priority areas (e.g., agriculture) also undermine utility of the current programmes (57). The lack of strong monitoring and evaluation mechanisms undermines the long-term impact of interventions (58). The difficulties of working with short-term projects hinder progress toward long-term sustainable solutions, and this results in disaggregated and non-effective strategies for nutrition security (59).

### Recommendations and way forward

Chronic and growing threats to nutrition security in Malawi hastened by recurring climate shocks, economic instability, and systemic inequities require a proactive, multisectoral approach that emphasises resilience, equity and sustainability. Projected scale-up As forecasted figures point to an expected increase in the number of people experiencing Crisis-level acute food insecurity (IPC Phase 3) to some 4 million from October 2025–March 2026, due to high food prices and persistent El Niño impacts, short- and longer-term action should build on gains already made such as the recently launched National Multisector Nutrition Policy and Strategic Plan (2025–2030) and identify key resource and coordination gaps. This 2025 June policy is another recommitment to reversing the malnutrition trends, however it depends on a K148 billion short fall in funding for the 2025–26 Lean Season Food Insecurity Response Programme. Drawing on insights from a recent evidence brief (designed to inform the next iteration of evaluation for nutrition security), as well as global best practices, there are 11 recommendations which detail strategies across specific domains with the ambition of enabling nutrition-secure futures by 2030, reducing stunting rates among children under five from their present levels at 38% and hence challenging entrenched cycles of vulnerability (1, 36).

Central to these initiatives should be strong (policy and institutional) reforms aimed at breaking down sectoral silos and enhancing the capacity of institutions. The Government of Malawi should lead the establishment of a National Nutrition Security Coordination Platform to bring together ministries of agriculture, health, finance and environment with non-governmental organizations and international partners like the World Food Programme and GIZ. Replicating the achievements of GIZ’s Food and Nutrition Security Programme (2015–2025), such a platform would facilitate coordinated policy implementation focused on resilience and social behavior change interventions. Pivotal actions include a proposed benchmark of progressively increasing investment toward approximately 10% of national budgets to nutrition-sensitive agriculture and climate adaptation by 2027 as well as passing gender-transformative laws ensuring women’s access to land and financial services addressing social norms that hinder household food distribution and child nutrition (51). In addition, integrating nutrition security within the post-2025 electoral agenda, with annual reporting mandatory against indicators from Sustainable Development Goal 2 will foster accountability and scale up, ensuring that the ambitious National Nutrition Policy results in actual stunting and wasting reduction.

In addition to such basic reforms, we must rapidly scale up agricultural and climate adaptation efforts if we are to keep Malawi’s rain-fed smallholder systems from being overwhelmed by mounting environmental challenges. Reports assessing the 2024 El Niño-related drought period estimated a substantial reduction in maize production, with national production declining to approximately 2.9 million metric tons. These estimates highlight the vulnerability of Malawi’s maize-dependent food system; however, the magnitude of production losses varies according to assessment methods, reference periods, and data sources (12). To reinforce food systems, preferential distribution at scale of subsidized drought resistant and biofortified varieties (e.g., vitamin A-enriched maize and iron-rich beans) to 80% of smallholders by 2028, should be accompanied by increased extension delivery (36, 37). Community-managed irrigation and agroforestry investments in the fickle rainfall belts of the south and centre (both flood- and drought-prone, having been affected by Cyclone Freddy) would alleviate soil degradation and the brunt of variable rainfall. Also critical to achieving that future is the launch of a digital early warning system and weather-indexed insurance at the national scale, in partnership with the Food and Agriculture Organization and through COMESA Climate Resilience Agriculture Development Support Programme, this will reduce post-harvest losses by 30 percent; reducing last month’s food inflation spike of 38.5percent observed in February 2025. This would not only help in diversifying production but also stabilizing markets, providing livelihood resilience to the 70% Malawians who are subsistence farmers to withstand future shocks more confidently indicating both economic and nutritional security (11).

Alongside progress on agriculture, nutrition-specific interventions need to address the underlying determinants of undernutrition, prioritising use and dietary diversity in disrupting intergenerational cycles. By building on the Malawi Nutrition Dialogues’ focus on solutions driven by community members, as well as universal micronutrient supplementation for pregnant and lactating women and behavior change communication for exclusive breastfeeding and complementary feeding, such interventions could reduce wasting from 10% to below 5% by 2030 (24, 30). Inclusion of therapeutic feeding protocols in primary health care services with regular screening for anemia and vitamin A deficiency in high-burden populations would protect children under age 5 and people living with HIV, because of their immunocompromised states, which increased risk of malnutrition (32). Incorporation of nutrition education, deworming and locally available nutrient rich foods in schools would also help offset cognitive losses due to stunting could allow for the formation of sustainable dietary patterns during these formative years with concomitant long-term gains in human capital (28). In the National Nutrition Policy, such targeted actions would reduce health burdens in vulnerable populations, especially the elderly—encouraging a healthier population during times of seasonal scarcity.

## Conclusion

Malawi’s nutrition security challenges are driven by the combined effects of climate shocks, dependence on rain-fed agriculture, economic vulnerability, and persistent social inequalities. This review highlights that climate-related disruptions affect nutrition outcomes through multiple pathways, including reduced agricultural productivity, increased food prices, declining household purchasing power, and limited dietary diversity. These challenges place vulnerable populations, particularly children under five, smallholder farmers, pregnant and lactating women, and people living with HIV, at increased risk of malnutrition.

Climate-smart agriculture, biofortification, social protection, and nutrition interventions provide important opportunities to strengthen resilience; however, their impact remains constrained by limited financing, fragmented implementation, and weak coordination. Addressing Malawi’s nutrition security requires integrated approaches that link climate adaptation, resilient food systems, health systems, and social protection. Future progress will depend on sustained investment, effective policy implementation, and coordinated multisectoral action.
